# Triphallia: the first cadaveric description of internal penile triplication: a case report

**DOI:** 10.1186/s13256-024-04751-5

**Published:** 2024-10-09

**Authors:** John Buchanan, Madeleine Gadd, Rose How, Edward Mathews, Andre Coetzee, Karuna Katti

**Affiliations:** University of Birmingham Medical School, Edgbaston, Birmingham, West Midlands B15 2TT UK

**Keywords:** Triphallia, Anatomy, Cadaveric dissection, Case report

## Abstract

**Introduction:**

Triphallia, a rare congenital anomaly describing the presence of three distinct penile shafts, has been reported only once in the literature. This case report, based on an extensive literature review, describes the serendipitous discovery during cadaveric dissection of the second reported human case of triphallia, distinctly morphologically different from the previous case.

**Case presentation:**

Despite the normal appearance of external genitalia on examination, the dissection of a 78-year-old white male revealed a remarkable anatomical variation: two small supernumerary penises stacked in a sagittal orientation posteroinferiorly to the primary penis. Each penile shaft displayed its own corpora cavernosa and glans penis. The primary penis and largest and most superficial of the supernumerary penises shared a single urethra, which coursed through the secondary penis prior to its passage through the primary penis. A urethra-like structure was absent from the smallest supernumerary penis.

**Conclusion:**

This case report provides a comprehensive description of the anatomical features of triphallia in a cadaver, shedding light on the morphology, embryology, and clinical implications of this anomaly. Without dissection, this anatomical variation would have remained undiscovered, suggesting the prevalence of polyphallia may be greater than expected. The single tortuous urethra present in this case, as well as the supernumerary and blind ending urethras present in many cases of penile duplication, may pose significant risk of infection, sexual dysfunction, subfertility, and traumatic catheterization.

**Significance:**

These findings underscore the importance of meticulous anatomical dissections and may act as a resource for anatomists and those studying genitourinary anomalies. Although we can only speculate as to which functional implications this patient may have experienced, understanding such anatomical variations contributes to both knowledge of human anatomy and clinical management should the condition be encountered in living individuals.

## Background

Congenital supernumerary penile formation is an extremely rare abnormality affecting approximately 1 in 5–6 million live births (1). In total, 168 papers, dated from 1606 to 2023, were found to report polyphallia (2, 3). According to the classification system by Vilanova (4), 112 showed complete diphallia, 50 demonstrated pseudodiphallia, and 1 reported triphallia; 5 did not contain sufficient information to classify. The phenotypic differences displayed across these papers varied dramatically, potentially due to the wide range of pathophysiological causes, however, this made little difference clinically. Almost all surgeons decided to remove an additional external penis, often the non-functional or smaller of the two. However, typically no action was taken with regard to internal penis formation, as they are usually asymptomatic.

Six diphallia cases have been reported with internal accessory penises, meaning that the accessory penis is concealed within the skin (5–10). Despite the majority of external penile duplication being associated with other congenital abnormalities, including midline structure duplication and vertebrae, anus, heart, trachea, esophagus, kidney, and limbs (VACTERYL) association, internal penile duplication often presents later in life as an incidental finding.

As the second known case of triphallia, this case is noteworthy, not only due to its rarity, but also because of the phenotypic differences between this case and the prior triphallia case reported by Jabali and Mohammed (1). As the inferior two penises were concealed within the scrotal sac, external genitalia appeared normal. This may explain why the abnormality was not observed until post mortem exploration.

Without any symptoms and additional medical needs, concealed internal penises may not present themselves, preventing diagnosis. Hence, polyphallia may be more prevalent than currently understood. It is of clinical importance for healthcare providers to be aware of polyphallia for the diagnosis of patients presenting with urological symptoms and for healthcare interventions, such as simple catheter insertion, urological imaging, and surgery.

This case report will provide a description of the triphallia, found incidentally, discussing its pathophysiology and potential impacts on health.

### Normal male genital embryology

From week 4 of embryonic development, there is proliferation of mesenchymal cells surrounding the cloacal membrane. At the caudal end, these form the genital tubercle, and at the cranial end, they form the urogenital folds (inner genital swellings) and the more lateral genital swellings (11, 12).

In fetuses with the Y chromosome, the sex-determining region Y (SRY) gene is expressed, triggering testicular development. This begins with the development of Sertoli cells, which induce development of Leydig cells. The Leydig cells produce testosterone from 8–9 weeks, which induces the development of male external genitalia (11, 13). Testosterone is converted to dihydrotestosterone (DHT) by 5α-reductase, which is expressed in the urogenital sinus and genital tubercle, and the DHT acts on androgen receptors in these areas to promote development of male external genitalia (13).

The genital tubercle elongates and becomes the corpora cavernosa and spongiosum, as well as the glans penis. The urethral plate (derived from endoderm) extends from the pelvic urethra into the glans penis. The urethral plate is canalized from proximal to distal, often referred to as the “opening zipper.” This canalization stops at the coronal sulcus, and hence does not extend into the glans penis. Urethral formation is different in the penile shaft as opposed to the glans penis. In the penile shaft, the edges of the canalized urethral plate fuse, forming the penile urethra (“closing zipper”), leaving the median penile raphe. In the glans there is direct canalization of the urethral plate to form the remainder of the urethra, with no urethral groove (13). The skin of the penis and the prepuce are formed from the ectoderm (11, 13).

## Case presentation

According to local policy, the identity of donors, and their medical history, must not be disclosed to researchers. As such, knowledge of this patient’s medical history is limited, and restricted to findings made during anatomical dissection and inspection.

### Patient description

This white male, in his late 70s, was around 6 feet tall and of a medium–large build. The most notable finding, as previously discussed, was the presence of internal penile triplication. These penile morphological abnormalities may not have been identified during his life. However, he may have lived with functional deficits due to the abnormal anatomy of the region, which may include urinary tract infections, erectile dysfunction, or fertility issues, as later described.

### Cadaveric findings

With the skin of the pelvis largely intact, the pelvis was divided in a midsagittal plane; we incised through the pubic symphysis, the penis, and the scrotum, and extended the dissection of the pelvic floor to just anterior to the anus. This midline dissection allowed us to appreciate a sagittal cross section of the penis as well as visualize both undissected testes, one on either side of the incision. Upon further exploration, three penis-like structures were found adjacent to each other, aligned in the mid-sagittal plane from dorsal to ventral. This is known as sagittal stacking (3). After further meticulous dissection, discussions and review of literature, we came to the conclusion that this was a case of triphallia.

**Fig. 1:**
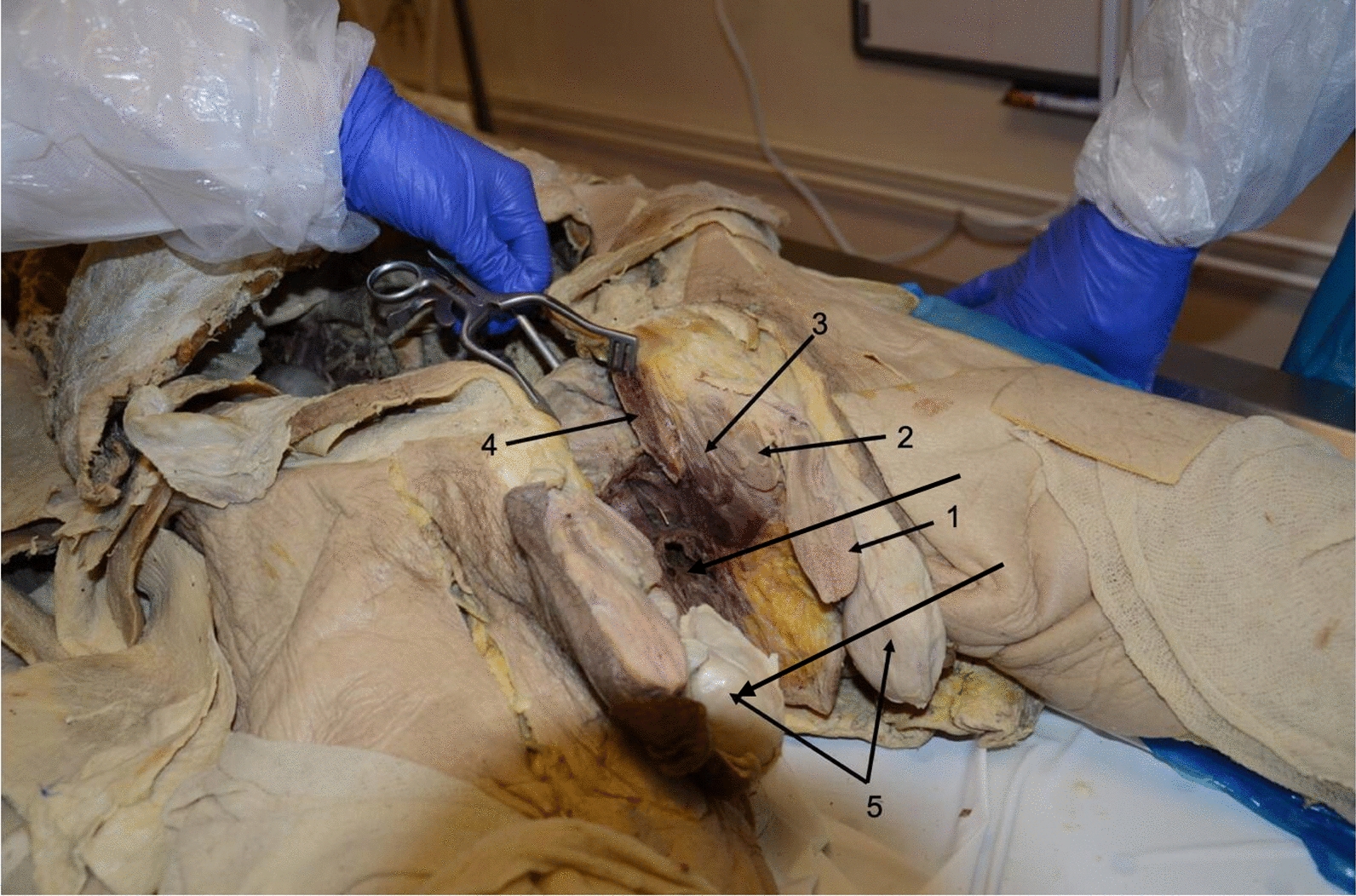
Sagittal, cross-sectional dissection of the male pelvis. This is a photograph taken of the male pelvis in situ, following a sagittal, cross-sectional, cadaveric dissection. The structures have been labelled as follows: 1. primary penis, 2. secondary penis, 3. tertiary penis, 4. pubic symphysis, and 5. testes. Following dissection in situ, the three penises along with the urinary bladder and prostate were excised together for further exploration

**Fig. 2:**
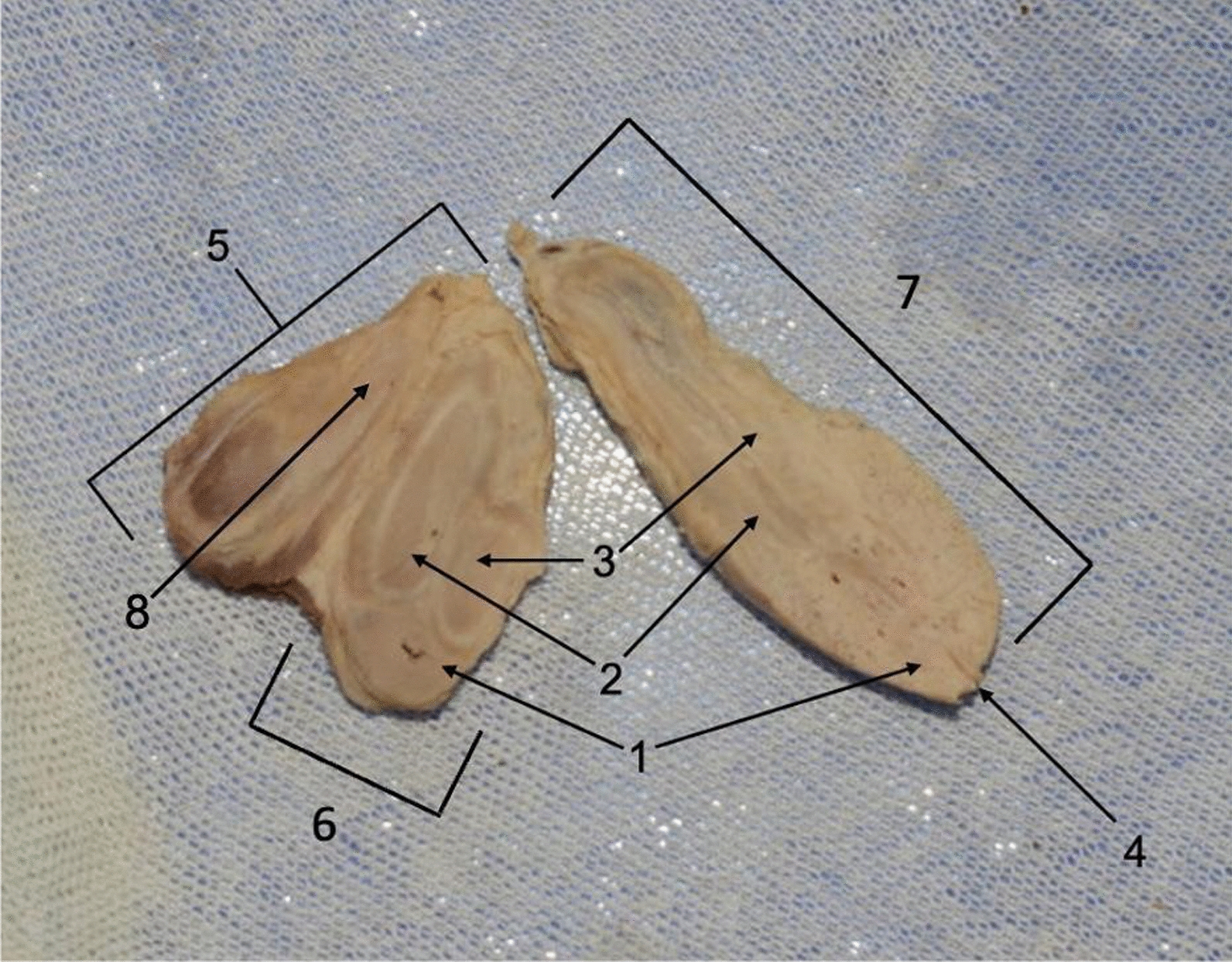
Ex situ triphallia dissection. This figure shows a midline section of the penile triplication. The following structures have been identified: 1. corpora spongiosum of glans penis (with urethra), 2. corpora spongiosa of primary and secondary penises, 3. corpora cavernosa of primary and secondary penises, 4. external urethral meatus of primary penis, 5. tertiary penis, 6. secondary penis, 7. primary penis, and 8. corpora cavernosa of tertiary penis

The largest and most dorsal of the structures was the only structure seen externally and hence will be referred to as the primary penis. The other two structures (termed the secondary and tertiary penises) appeared to be located within the skin of the scrotal sac, hence why they were not visible in the undissected specimen. The secondary penis was located immediately deep to the primary penis and had macroscopically discernible and distinct regions comparable to the normal penile anatomy (the corpus spongiosum and urethra, corpora cavernosa, and the glans penis). The tertiary penis lay deep to the secondary penis, however, did not have the same obvious anatomical features seen in the primary or secondary penises; corpora cavernosa and glans penis were identified, however, there was an absence of corpus spongiosum and a urethra (Figs. 1, 2).

With the aim of tracing the urethra of the primary penis, a probe was inserted into the external urethral orifice and advanced into the urethra. When it became apparent that the probe could not be advanced further, the primary penis was dissected more posteriorly in a sagittal plane. At this point, it became evident that the urethra of the primary penis extended into the secondary penis, and then could be traced superiorly to the internal urethral orifice. This revealed the meandering course of a single urethra, devoid of any branches, through the secondary and then primary penis up to the external urethral orifice. No urethra-like structure was identified in the tertiary penis.

Some important negative cadaveric findings in this case are accessory kidney, ureter, or bladder; duplication of the lower gastrointestinal tract; bifid or solitary scrotum; imperforate anus; and anterior abdominal wall defects.

**Fig. 3:**
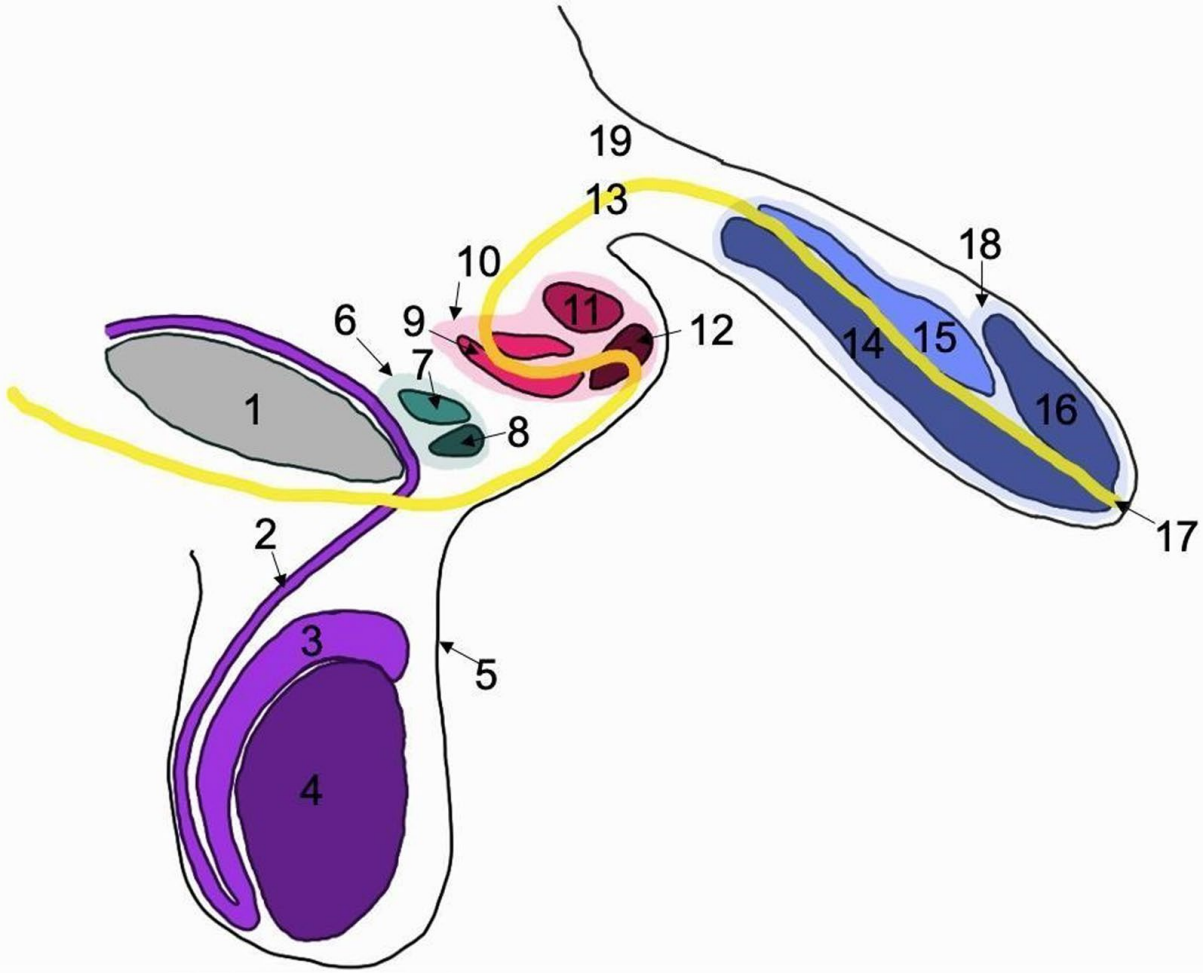
Schematic diagram of internal triphallia. This figure is a schematic diagram of the sagittal cross section of the male pelvis in this case. The arrangement of triphallia can be seen above. The diagram can be labeled as follows: 1. pubic symphysis, 2. vas deferens, 3. epididymis, 4. testicle, 5. skin, 6. tertiary penis, 7. corpus cavernosum of tertiary penis, 8. glans of tertiary penis, 9. corpus spongiosum of secondary penis, 10. secondary penis, 11. corpus cavernosum of secondary penis, 12. glans of secondary penis, 13. urethral pathway, 14. corpus spongiosum of primary penis (continuous with glans), 15. corpus cavernosum of primary penis, 16. glans of primary penis, 17. urethral opening, 18. primary penis, and 19. connective tissue

The schematic diagram below (Fig. 3) shows the primary penis and two supernumerary penises, the penile and scrotal skin, and the testis.

The dimensions of the three penile structures are presented in Table 1.

**Table 1:** Penile dimensions

	Length (mm)	Width (mm)
Primary penis	77	24
Secondary penis	38	13
Tertiary penis	37	12

This table lists the dimensions of each penis. All measurements of length were taken from the base of the body of the penis to the furthest point at the tip of the glans penis. All measurements of width were taken at the widest point of the glans penis from dorsal to ventral.

## Discussion

The penis develops from the genital tubercle and is controlled by DHT. Genetic abnormalities affecting the expression of androgen receptors may cause morphological genital abnormalities (1). In this case, there may have been triplication of the genital tubercle. The urethra originally developed in the secondary penis, however, when this penis failed to develop, the urethra diverted its course and developed in the primary penis instead. The tertiary penis is a remnant of the triplicated genital tubercle.

There is an increased risk of urinary tract infections in diphallia or triphallia cases with multiple or blind-ending urethras due to urine stagnation (14). In this case, the risk of urinary tract infections (UTIs) is not likely to be increased as there is no blind-ending urethra. This person may have experienced dyspareunia due to the potential erection of the secondary and tertiary penises.

Most cases of supernumerary penises either presented in the neonatal period with evident external polyphallia or with sexual dysfunction, obstructive urinary symptoms, and urinary incontinence in adulthood (2, 8, 15–17). The majority of these cases are associated with various other congenital defects, including “defects in number” of kidneys, ureters, and bladders; ectopic and extrophic bladder and other abdominal viscera; supernumerary or imperforate anuses; and hypo/epispadias; among many others (6, 18–22).

We cannot be certain that in this case the defect remained unnoticed in life, as there is a history of inguinal hernia repair. Due to the tortuous nature of the urethra, a urinary catheter would have proved challenging to pass. If the defect had been noticed during his life it may have simply been left untouched due to the apparent lack of symptoms and its benign nature. In other similar cases of supernumerary penises, but with prevalent undesirable symptoms or cosmetic appearance, surgical resection was frequently utilized (23–26).

There are currently no all-encompassing or clinically useful classification systems for supernumerary penises. The majority of studies use the classification system proposed by Schneider *et al*. (27); however, their categories fail to involve all cases of supernumerary penises and have little clinical relevance. Other systems have been created, such as that produced by Kendrick and Kimble (3), which builds on the Schneider classification. This newer system, despite capturing a greater number of the phenotypes and describing them more clearly, adds in a degree of complexity that once again reduces the clinical usefulness of the system. Moreover, due to the uncertainty surrounding nomenclature in the literature, classification of this case is difficult.

## Conclusion

Through lack of consensus and the poor clinical usefulness of the current systems, a new classification system that retains simplicity and uniformity would be invaluable in the clinical setting and should be the focus of further reports.

## Data Availability

Not applicable.

## Abbreviations

SRY: Sex-determining region Y
DHT: Dihydrotestosterone
UTI: Urinary tract infection
